# Specialities and Recently Introduced Remedies

**Published:** 1889-09

**Authors:** 


*Specialities and Recently Introduced Remedies.* Manchester: JAMES WOOLLEY, SONS & CO. 1889.

We have been pleased at receiving this tastefully got-up list, which should be kept well within reach on the consulting-room table, and which, doubtless, Messrs. Woolley will readily send to any practitioner asking for it.

